# Cerebral oximetry monitoring in pediatric seizure patients in the emergency department

**DOI:** 10.1186/cc14539

**Published:** 2015-03-16

**Authors:** T Abramo, B Schnieder, E Storm, N Hobart-Porter, Z Hu, N Todd, L Crawley, M Meredith, S Godbold

**Affiliations:** 1 University of Arkansas School of Medicine, Little Rock, AR, USA; 2 University of Tennessee School of Medicine Memphis, TN, USA

## Introduction

During ictal/post-ictal events, altered cerebral physiology occurs: increased neuronal activity causes significant increase in cerebral metabolism with changes in ipsilateral cerebral blood flow. Standard PED seizure monitoring is by O_2_SAT and ETCO_2_ which yield no direct data about regional cerebral oxygenation/physiology (rSO_2_). Significant abnormal hemispheric cerebral physiology resulting in neurological injury can occur without knowing because the current monitoring system could not detect the abnormal hemispheric abnormality. Cerebral oximetry can provide a rapid, non-invasive detection of each hemisphere's cerebral physiologic changes during ictal/post-ictal phases. The aim was to study left and right rSO_2_ values in patients in the pre and post seizure periods and in nonseizing controls.

## Methods

An observational study of seizing and nonseizing patient's left and right rSO_2_ readings compared with nonseizure patients.

## Results

**Figure 1:**
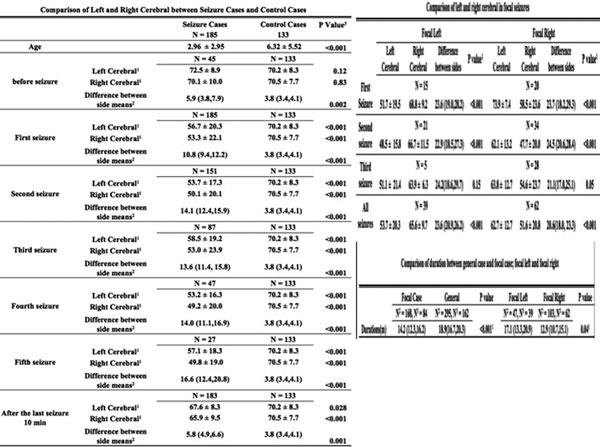


No difference for ictal left and right rSO_2_ readings across ages. See Figure 1.

## Conclusion

We have demonstrated abnormal hemispheric cerebral physiology during focal or generalized ictal activity. In patients with generalized seizures, the left and right rSO_2_ values were significantly decreased. In patients with focal seizures, the ipsilateral rSO_2_ values were significantly different from the contralateral rSO_2_ readings and correlated to the hemisphere experiencing the focal seizure. In certain patients, during the ictal phase their rSO_2_ readings rose and stayed or rose then dropped. Overall, cerebral oximetry has shown great monitoring potential for actively seizing patients in the emergency department.

